# The effects of fucoidan as a dairy substitute on diarrhea rate and intestinal barrier function of the large intestine in weaned lambs

**DOI:** 10.3389/fvets.2022.1007346

**Published:** 2022-10-20

**Authors:** Guangzhen Guo, Weiguang Yang, Chaojie Fan, Ruixia Lan, Zhenhua Gao, Shangquan Gan, Haibin Yu, Fuquan Yin, Zhijing Wang

**Affiliations:** ^1^College of Coastal Agriculture Science, Guangdong Ocean University, Zhanjiang, China; ^2^The Key Laboratory of Animal Resources and Breed Innovation in Western Guangdong Province, Department of Animal Science, Guangdong Ocean University, Zhanjiang, China; ^3^Animal Disease Prevention and Control Center, Guangdong Qingyuan Agricultural Bureau, Qingyuan, China

**Keywords:** cecal short-chain fatty acids, cecal microbes, colon histomorphology, colon antioxidants, colon immunity, colon cytokines

## Abstract

This paper explores the effects of fucoidan on the frequency of diarrhea, colon morphology, colon antioxidant status, cytokine content, short-chain fatty acids, and microflora of cecal contents in early weaned lambs in order to provide a reference for the intestinal health of young ruminants. Fucoidan is a natural active polysaccharide extracted from kelp and other large brown algae. It has many biological effects, such as improving immunity, nourishing the stomach and intestines, and anti-tumor properties. This study investigated the effects of fucoidan supplementation in milk replacer on the large intestine's ability to act as an intestinal barrier in weaned lambs. With six duplicate pens and one lamb per pen, a total of 24 weaned lambs (average starting body weight of 7.32 ± 0.37 kg) were randomly assigned to one of four milk replacer treatments. Four concentrations of fucoidan supplementation (0, 0.1, 0.3, and 0.6% dry matter intake) were employed to investigate the effects of fucoidan on cecal fermentation and colon microbial organization. The test period lasted 37 days (1 week before the test and 1 month after the test), and lamb cecal contents and colon organization were collected for examination. In addition, the fecal status of all lambs was observed and recorded daily, allowing us to calculate the incidence of diarrhea in weaned lambs. The findings demonstrated that fucoidan may significantly increase the concentration of short-chain fatty acids (propionic acid and butyric acid) in the cecal digesta of weaned lambs. In weaned lambs, 16S rDNA testing showed that fucoidan at 0.3–0.6% (dry matter intake) was beneficial for boosting the variety of the intestinal bacteria and modifying the relative abundance of a few bacterial strains. In addition, fucoidan enhanced colon antioxidant and immune functions and decreased the diarrhea rate to relieve weaning stress. This result demonstrates that milk replacer supplementation with fucoidan contributes to the improvement in the large intestinal health of weaned lambs.

## Introduction

The number of sheep and goats in China ranks among the highest in the world, with an estimated 307 million domestic sheep and goats (1). However, goat husbandry in southern China is influenced by factors such as geographic location, climatic conditions, and breed. Early weaning is a crucial technological advancement. It can lead to the growth of the lamb and shorten the ewes' breeding and estrous cycles (2). Unfortunately, early lamb weaning technology remains underexploited because the digestive systems of lambs are immature and cannot take maximal advantage of plant-based solid feed. In addition, weaning is a critical phase of well-marked physiological change and evokes psychological and physiologic stress responses, especially post-weaning diarrhea (PWD), which is a common problem in lambs (3–5). These challenges restrict the further development of the industry. Therefore, novel nutritional approaches to alleviate weaning stress have become a major focus of industry interest in young animal health, breeding, and feed.

In recent years, biologically active substances extracted from plants have attracted increasing attention for the prevention and treatment of diseases. Seaweed polysaccharides have been shown to improve immune responses, redox status, and gut health in animals (6–10). Fucoidan refers to a group of complex sulfated polysaccharides derived from brown seaweed species. Its core components are fucose and sulfate groups, which mediate a variety of significant biological effects with antioxidant (11), anti-inflammatory (12, 13), and immune-enhancing (14–18) properties. These properties can alleviate diarrhea symptoms and improve growth (19). Fucoidan, as a natural strong antioxidant, can effectively remove free radicals, reduce the production of inflammatory factors, and alleviate inflammatory reactions (11–13). At the same time, the alternating (1 → 3) and (1 → 4) glycosidic bonds on the main chain of fucoidan have a regulatory effect on intestinal immunity. The intestinal immune balance is maintained by stimulating a variety of mononuclear cells, macrophages, and auxiliary T-cells, as well as regulating the secretion of cytokines such as TNF-α (14–18). O'Shea (19) found that after feeding 240 mg/kg fucoidan to piglets affected by chronic colitis, diarrhea symptoms were significantly improved and weight gain was obvious.

We previously showed that lambs fed fucoidan had an average daily weight gain that was increased by over 34% over 30 days compared to the control group that did not receive fucoidan. We found that feeding fucoidan at concentrations of 0.3 and 0.6% improved antioxidant enzyme activity, raised the amount of anti-inflammatory substances in blood samples from weaned lambs, and decreased the amount of pro-inflammatory substances (20). However, there is no evidence to explain the mechanism by which fucoidan alleviates PWD in lambs. Therefore, this paper explores the effects of fucoidan on the frequency of diarrhea, colon morphology, colon antioxidant status, cytokine content, short-chain fatty acids, and microflora of cecal contents in early weaned lambs in order to provide a reference for the intestinal health of young ruminants.

## Materials and methods

### Materials

The fucoidan was purchased from Mingyue Hailin Fucoidan Biotechnology Co., Ltd. (Qingdao City, Shandong Province, China). The fucoidan was produced on January 5, 2021, with the following characteristics: purity: 98%, appearance: light yellow powder, composition: 66.3% sugar, 24.9% fucose, and 28.9% sulfate, and moisture level: 7.87%. The milk replacer power was obtained commercially (Beijing Precision Animal Nutrition Research Center, Beijing, China). The starter was formulated to meet or exceed the recommendations for lamb nutrition under China's Agricultural Industry Standard (Concentrate:Roughage = 80:20; NY/T 816-2004, Table 1).

**Table 1:** Composition and nutrient composition of the starter (dry matter basis) %.

**Ingredient**	**Content**	**Nutrition level**	**Starter content**	**Milk replacer power content**
Alfalfa meal	23.16	DM	89.25	97.58
Expanded soybean	32.97	DE^2^/(MJ/kg)	13.45	11.14
Corn	22.49	CP	23.20	15.40
Rapeseed meal	7.33	EE	8.14	15.43
Bran	3.71	Ash	5.06	14.52
Cottonseed cake	7.33	Ca	0.92	1.02
Limestone	0.50	P	0.58	0.66
NaCl	0.52	NDF	17.15	
CaHPO_4_	1.00	ADF	5.45	
Premix^1^	1.00			
Total	100.00			

^1^The premix provided the following per kg of diet: Fe 1.10 g, Cu 0.73 g, Mn 0.31 g, Zn 0.26 g, I 0.01 g, Se 0.02 g, Co 0.22 g, vitamin A 76 190 IU, vitamin D 3 429 IU, vitamin E 170 IU, vitamin B_1_ 23.32 mg, vitamin B_2_ 28.00 mg, vitamin B_6_ 22.63 mg, vitamin B_12_ 137.13 mg, vitamin B_5_ 91.42 mg, nicotinic acid 181.01 mg. ^2^Digestive energy was a calculated value, while the others were measured values.

### Animals, experimental design, and management

The experiments were performed under the supervision of Guangdong Ocean University's Animal Care and Use Committee. 24 healthy Chuanzhong black male lambs (30 days old) with equal body weights of 7.32 ± 0.37 kg were weaned and provided with unrestricted access to water, starter food, and milk replacer powder for at least 7 days. Lambs were randomized into four groups (control, low-dose, middle-dose, and high-dose; *n* = 6 each). The lambs in the low-dose group (FL) received fucoidan at 0.1% of their daily dry matter intake (DMI). For the middle-dose group (FM), lambs were given fucoidan at a daily dose of 0.3% DMI. The lambs in the high-dose group (FH) were given fucoidan at a daily dose of 0.6% DMI.

All lambs were freely fed starters, and an equal amount of milk replacer powder (1.2%) was provided on a daily basis at 08:00, 11:00, 14:00, and 17:00 h. The milk replacer powder containing fucoidan was reconstituted with boiling water (weight of milk replacer powder: volume of water = 1:6). All lambs were single-housed. The total test period was 37 days. Lamb housing was cleaned daily, disinfected, and ventilated regularly. On the last day of the study, the lambs were sacrificed by jugular exsanguination. The cecum contents and colon tissues were then removed separately and immediately stored at −80°C until needed.

### Fecal index and diarrhea rate

**Figure 1:**
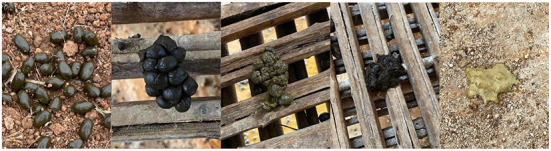
Fecal consistency score [**(left–right)**: score of 1, 2, 3, 4, and 5].

The fecal status of all lambs was observed and recorded on a daily basis at 08:00, 13:00, and 18:00 h. A fecal score of > 3 was considered diarrhea. Figure 1: Fecal scoring standards were: 1, hard and well-formed pellets; 2, normal and formed pellets; 3, pasty and semi-formed pellets; 4, soft and pasty stools; 5, watery diarrhea. The fecal index and diarrhea rate were calculated as follows: Fecal index = total fecal score / all lambs; diarrhea rate = frequency of diarrhea in each group / (test days × total animal numbers in each group) (21).

### Colon histomorphology

Colons were postfixed for 24 h in 4% paraformaldehyde and processed in paraffin. The wax blocks were then divided into 4 μm chunks using a microtome, then dewaxed. The sections were deparaffinized in two changes of xylene before being rehydrated in ethanol. Hematoxylin-eosin staining (HE) and diastase-periodic acid-Schiff (D-PAS) staining were performed with kits (Servicebio, Wuhan, China) in accordance with the manufacturer's instructions. Histologic structures were observed using an inverted fluorescence microscope. Villus height, crypt depth, mucosal thickness, and muscular thickness were determined.

### Colon antioxidant and immune function

The colon tissues were removed from the −80°C freezer and slowly thawed at 4°C. Colon tissues of each group were accurately weighed to achieve a weight (g):volume (mL) ratio of 1:9. An homogenizer was used to homogenize and centrifuge the samples using 0.9% normal saline as the diluent. Biochemical assays were performed according to the manufacturers' kit instructions. Assay kits for the total antioxidant capacity (T-AOC), activities of superoxide dismutase (SOD), catalase (CAT), and glutathione peroxidase (GSH-Px), and malondialdehyde (MDA) content were purchased from the Nanjing Jiancheng Bioengineering Institute (Nanjing, China). Enzyme-linked immunosorbent assay (ELISA) kits for IL-10, IL-1β, TNF-α, IFN-γ, IgA, IgG, IgM, and SIgA were obtained from Jiangsu Meimian Industrial Co., Ltd. (Nanjing, China).

### Cecum microflora 16S rDNA sequencing and analysis

The manufacturer's procedure was followed to obtain genomic DNA. After quality and purity assessments, the extracted DNA samples were used as PCR templates. The obtained DNA bands were visualized by 1.8% agarose gel electrophoresis. The 16S rDNA V3 + V4 region of the sample was then amplified using primers 338F (5'-ACTCCTACGGGAGGCAGCA-3') and 806R (5'-GGACTACHVGGGTWTCTAAT-3') and enriched by PCR amplification to form a final library. All sequencing was conducted by Biomarker Technology Co., Ltd. (Beijing, China).

### Cecum short-chain fatty acids

The content and composition of cecal SCFA were determined using gas chromatography-mass spectrometry (Trace 1310 and ISQLT, Thermo, USA) following previously published protocols (22–24). The following chromatographic settings were utilized: injection volume of 1 μL; inlet temperature of 250°C; split ratio of 4:1; ion source temperature of 300°C, and transfer line temperature of 250°C. Oven temperature program: starting temperature at 90°C, then 10°C/min to 120°C and 5°C/min to 150°C, followed by a 25°C/min, 2 min climb to 250°C. Helium was used as the carrier gas, at a flow rate of 1.0 mL/min. The following mass spectrometry conditions were used: the electron ionization source of the instruments was operated with an electron energy of 70 eV and a SIM scanning mode was adopted. Recoveries and SCFA content were calculated following the method described by Giera et al. (25).

### Statistical analysis

The data were subjected to one-way ANOVA using SPSS 23.0 (SPSS, Inc., Chicago, IL, United States) to analyze the effects of fucoidan. Significant differences between means were compared using Duncan's multiple comparisons test. The standard error of the mean (SEM) was used to represent the variation. Prism 8 was used to plot diarrhea rates, colon tissue shape, antioxidant and immunological markers, and cecal short-chain fatty acid concentrations of the lambs, which were deemed significant at *P* < 0.05 (GraphPad Software, San Diego, CA, USA).

## Results

### The effect of fucoidan on diarrhea rate and fecal index in weaned lambs

**Figure 2:**
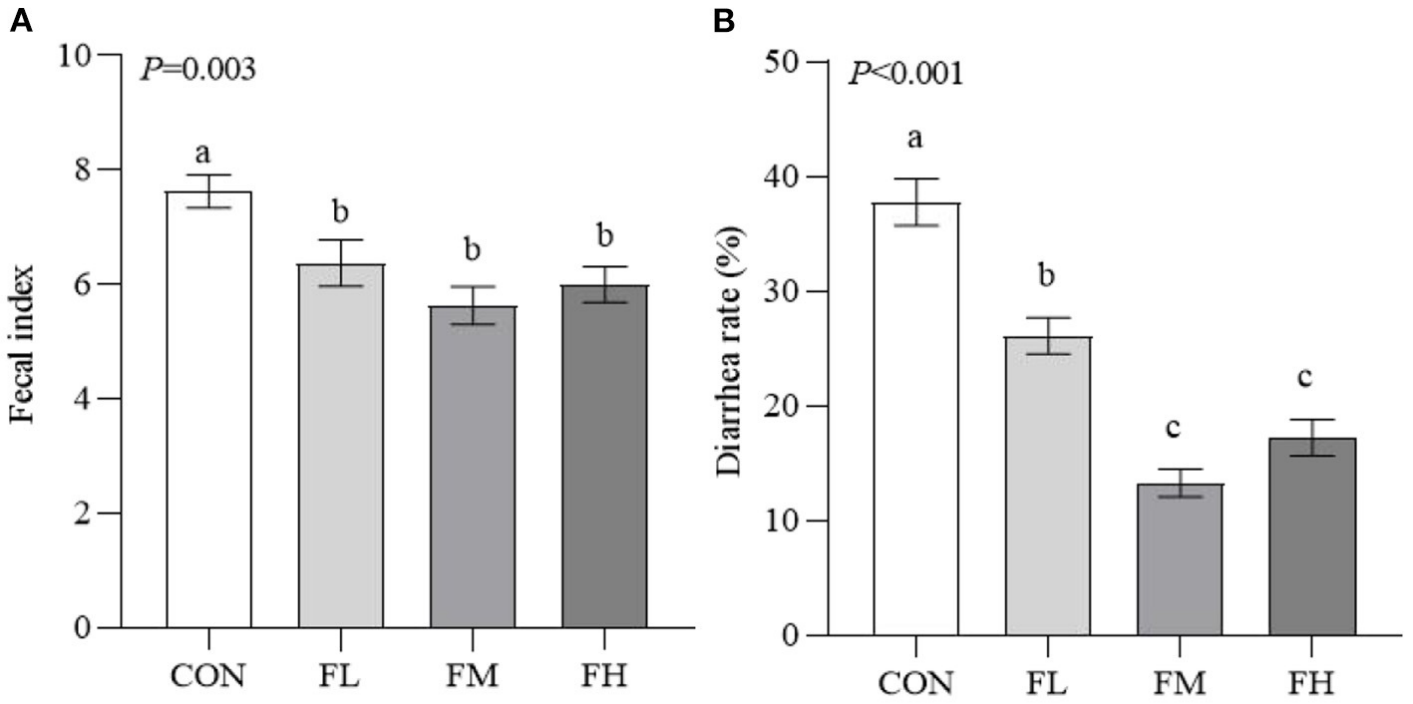
Effects of fucoidan on the fecal index and diarrhea rate of weaned lambs. **(A)** Fecal index. **(B)** Diarrhea rate. CON, control; FL, 0.1% fucoidan (DMI); FM, 0.3% fucoidan (DMI); FH, 0.6% fucoidan (DMI). Data are denoted as mean ± standard error of the mean (SEM) (*n* = 6). ^a,b,c^Demonstrate a statistically significant distinction, *P* < 0.05.

The fecal index and diarrhea rate reflect the health of the intestines, which impacts the growth of the weaned animals (Figure 2). Compared to the control group, the fucoidan-treated lambs had considerably lower fecal indexes and diarrhea rates (*P* < 0.05). The fecal index and diarrhea rate in groups FL, FM, and FH were significantly lower than those in the control by 16.38, 26.21, and 21.36% (*P* < 0.05) and 30.89, 64.85, and 54.42% (*P* < 0.001), respectively. However, the fecal index was not decreased in a dose-dependent manner when the weaned lambs were fed fucoidan.

### The effects of fucoidan on the colon histomorphology of weaned lambs

**Figure 3:**
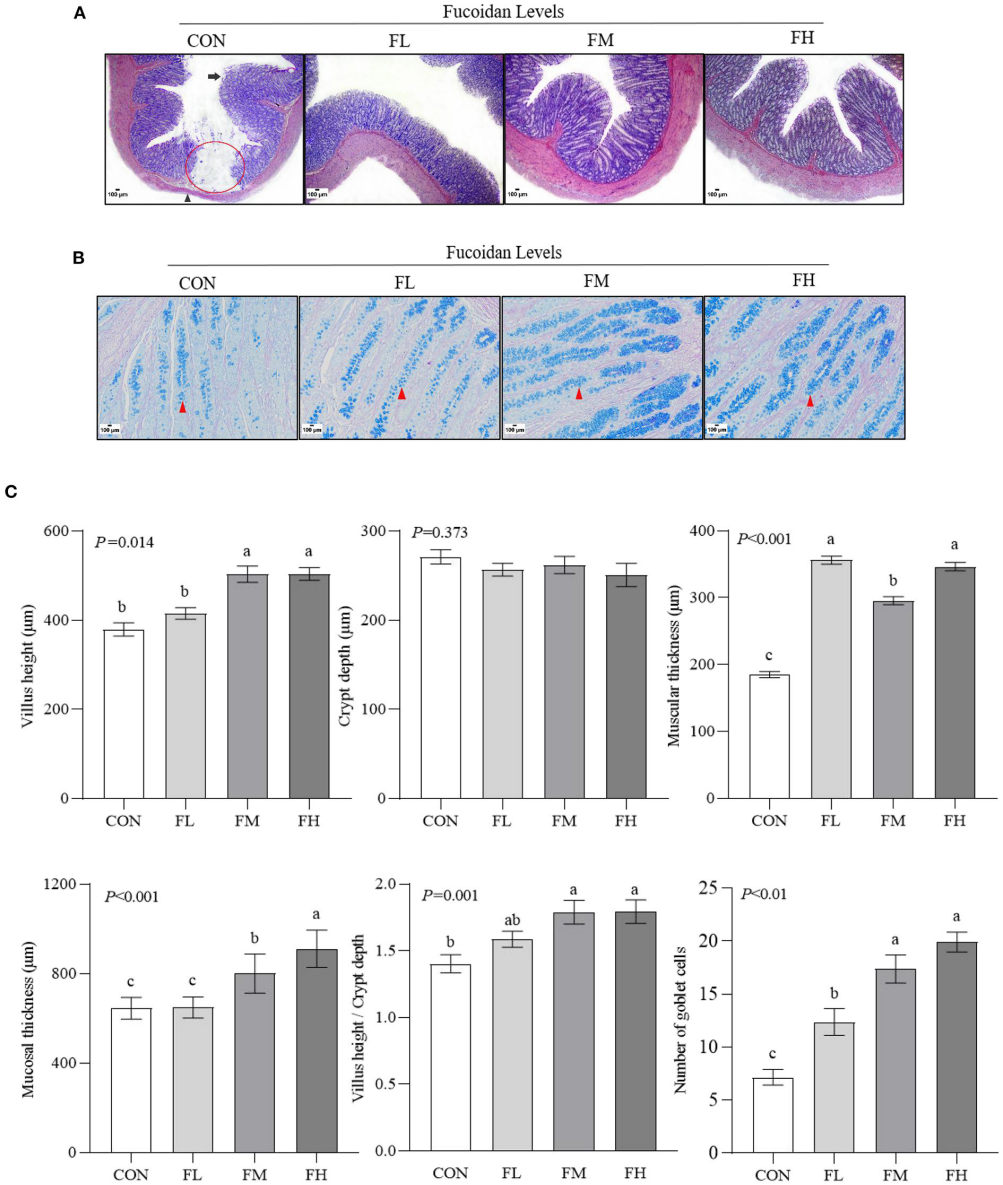
The effects of fucoidan on the colon morphology and goblet cell count of weaned lambs (*n* = 3). **(A)** HE staining results of colon paraffin sections (40×). The red circle indicates the absence of intestinal mucosa; the long black arrow shows the shedding of mucosal epithelial cells; the black arrowhead shows muscular atrophy. **(B)** D-PAS staining results of colon paraffin sections (200 × ). The red arrowhead shows the goblet cell (blue). **(C)** Morphology and goblet cell number in lamb colons were analyzed. CON, control; FL, 0.1% fucoidan (DMI); FM, 0.3% fucoidan (DMI); FH, 0.6% fucoidan (DMI). Data are denoted as mean ± SEM (*n* = 3). ^a,b,c^Demonstrate a statistically significant distinction, *P* < 0.05.

To assess the histological changes in the goblet cells of the colon in the lambs, we performed HE staining (Figure 3A). Meanwhile, the status of mucin-producing epithelial goblet cells was evaluated through D-PAS staining (Figure 3B). The results were confirmed *via* software analysis (Figure 3C). Compared with feeding 0.3~0.6% fucoidan, the villus height and mucosal thickness in the CON and FL groups were markedly decreased (Figure 3C) (*P* < 0.05), and the villus epithelial cells were shedding in the CON (the long black arrows show the shedding of mucosal epithelial cells; Figure 3A). In contrast to the control group, fucoidan-fed lambs had increased numbers of colon goblet cells (the red arrowheads show the goblet cells; Figure 3B), increased ratio of villous height to crypt depth (Figure 3C), and increased thickness of the colon muscular walls, especially in the 0.3~0.6% fucoidan groups (Figures 3A,C; *P* < 0.05).

### The effects of fucoidan on colon antioxidant in weaned lambs

**Figure 4:**
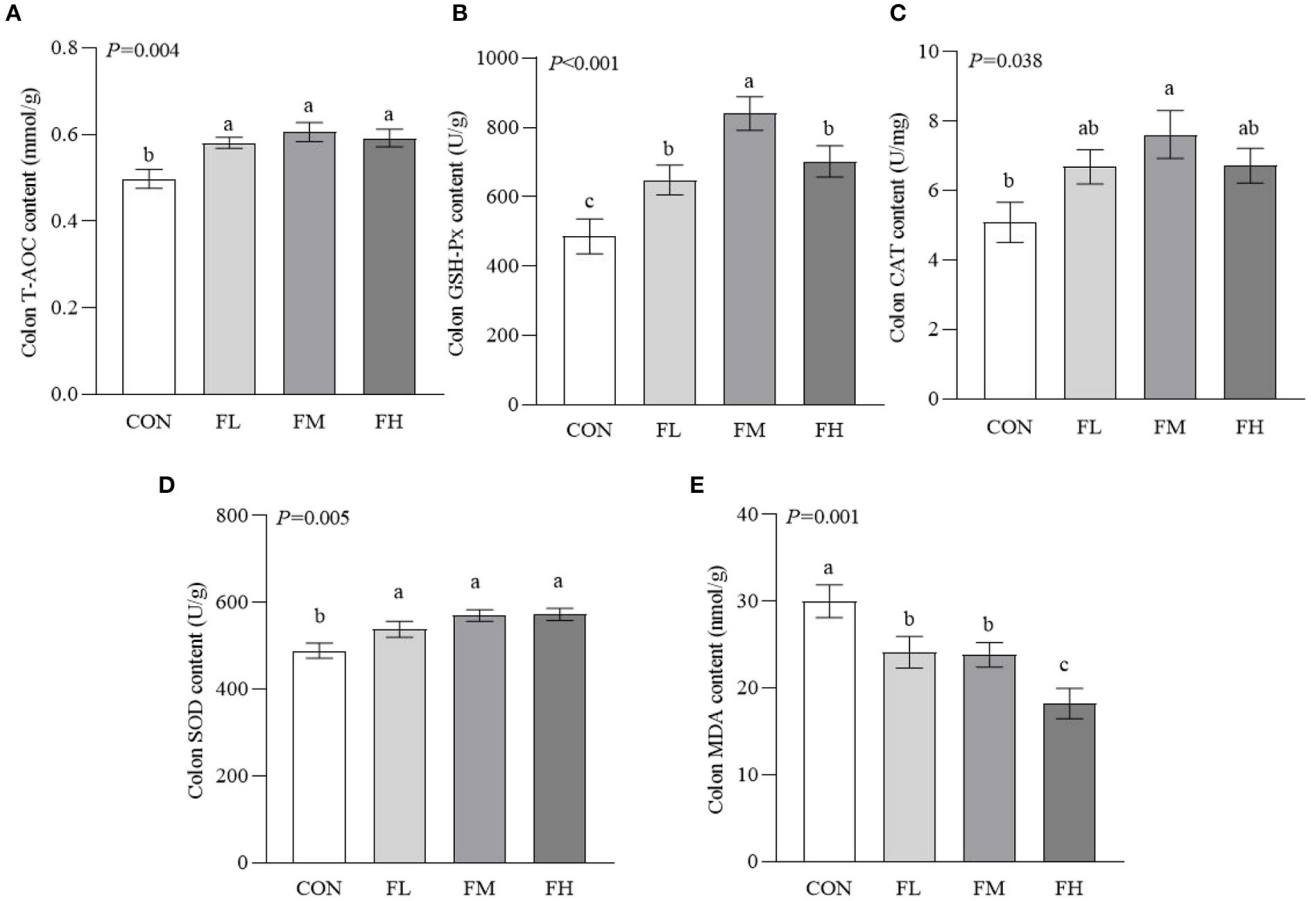
Effects of fucoidan on the colon antioxidant indexes of weaned lambs. **(A)** T-AOC. **(B)** GSH-Px. **(C)** CAT. **(D)** SOD. **(E)** MDA. CON, control; FL, 0.1% fucoidan (DMI); FM, 0.3% fucoidan (DMI); FH, 0.6% fucoidan (DMI). Data are denoted as mean ± SEM (*n* = 3). ^a,b,c^Demonstrate a statistically significant distinction, *P* < 0.05.

To determine how fucoidan affects the colon antioxidant system, antioxidant enzyme activities were measured (Figure 4). The T-AOC and the activities of GSH-Px and SOD were higher in the fucoidan-treated groups than in the CON group (*P* < 0.05), particularly the FM group. The CAT activity of the FM group was significantly higher than in the CON group (*P* = 0.005). Meanwhile, the MDA content of the CON group was greater than those of the groups that were fed fucoidan (*P* = 0.001).

### The effects of fucoidan on colon immunity in weaned lambs

**Figure 5:**
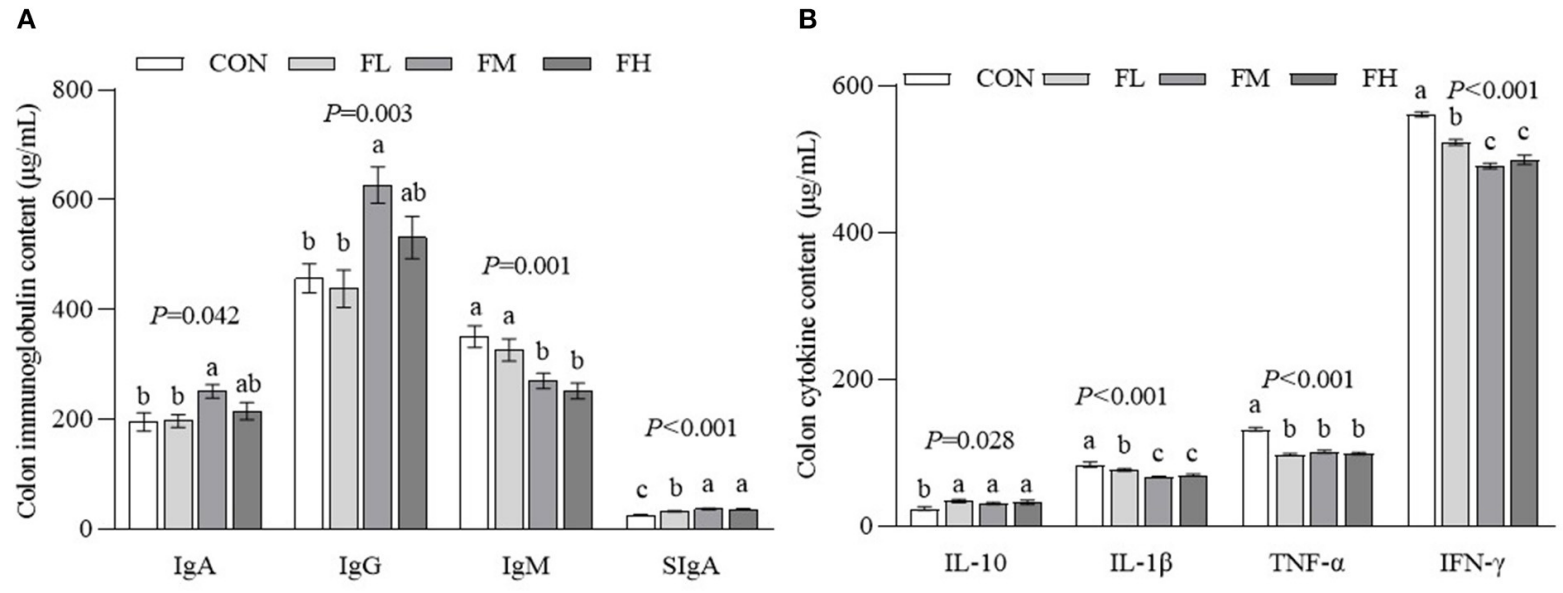
The effects of fucoidan on colon immunoglobulin and cytokine content in weaned lambs. **(A)** Colon immunoglobulin concentrations. **(B)** Colon cytokine concentrations. CON, control; FL, 0.1% fucoidan (DMI); FM, 0.3% fucoidan (DMI); FH, 0.6% fucoidan (DMI). Data are denoted as mean ± SEM (*n* = 3). ^a,b,c^Demonstrate a statistically significant distinction, *P* < 0.05.

The gut is the largest immune organ in the body. We evaluated the colon immunoglobulin and cytokine contents through ELISA (Figure 5). Compared with the CON and FL groups, the contents of IgA and IgG in the FM group were higher (*P* < 0.05). The SIgA contents of the fucoidan-fed groups were considerably greater (*P* < 0.05) compared to controls, particularly the FM and FH groups. The CON and FL groups had more IgM than the FM and FH groups (*P* < 0.05). The CON group also had a greater proinflammatory factor content than the fucoidan-fed groups (*P* < 0.05), especially the FM and FH groups. Although the IL-10 concentration in the CON group was greater compared to the fucoidan-fed groups (*P* < 0.05), there was no obvious dose-dependent effect (*P* > 0.05).

### The effects of fucoidan on cecum SCFA content in weaned lambs

**Figure 6:**
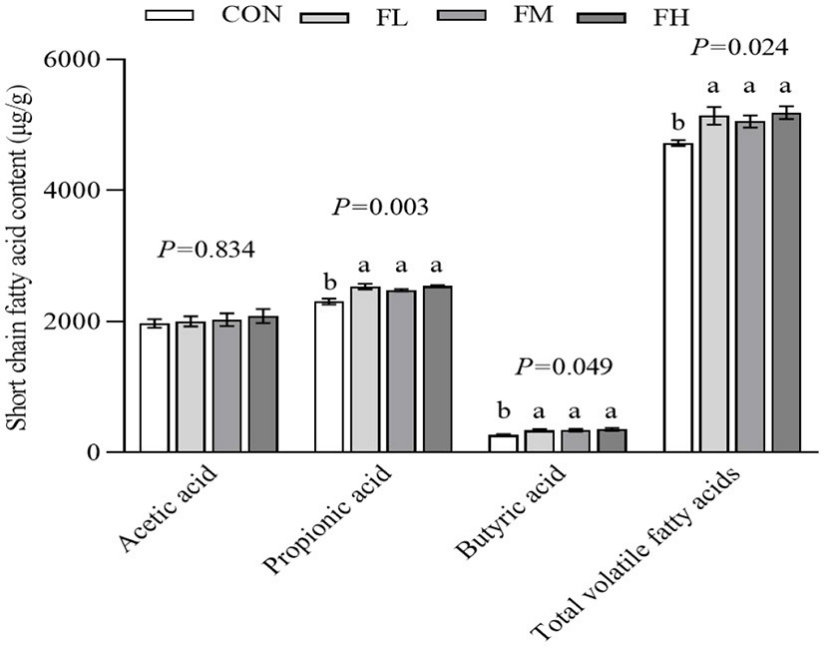
The effects of fucoidan on cecum SCFA in weaned lambs. CON, control; FL, 0.1% fucoidan (DMI); FM, 0.3% fucoidan (DMI); FH, 0.6% fucoidan (DMI). Data are denoted as mean ± SEM (*n* = 3). ^a,b^Demonstrate a statistically significant distinction, *P* < 0.05.

The content and composition of cecum SCFA were determined using gas chromatography-mass spectrometry (Figure 6). The fucoidan-fed groups had greater levels of propionic acid, butyric acid, and total volatile fatty acids than the CON group (*P* < 0.05), but there was no obvious dose-dependent impact (*P* > 0.05).

### The effects of fucoidan on gut bacterial composition in weaned lambs

By sequencing the bacterial 16S rDNA V3 + V4 region, the microbiota of the cecal contents in the four groups of weaned lambs were examined. High-throughput sequencing was conducted on three random cecum samples from each group. 18 samples produced a total of 956,804 clean reads, with 79,734 clean reads on average (*n* = 3). These sequences were assigned to 28 phyla, 52 classes, 116 orders, 216 families, 459 genera, and 528 species based on a 97% similarity definition of an operational taxonomic unit (OTU).

**Figure 7:**
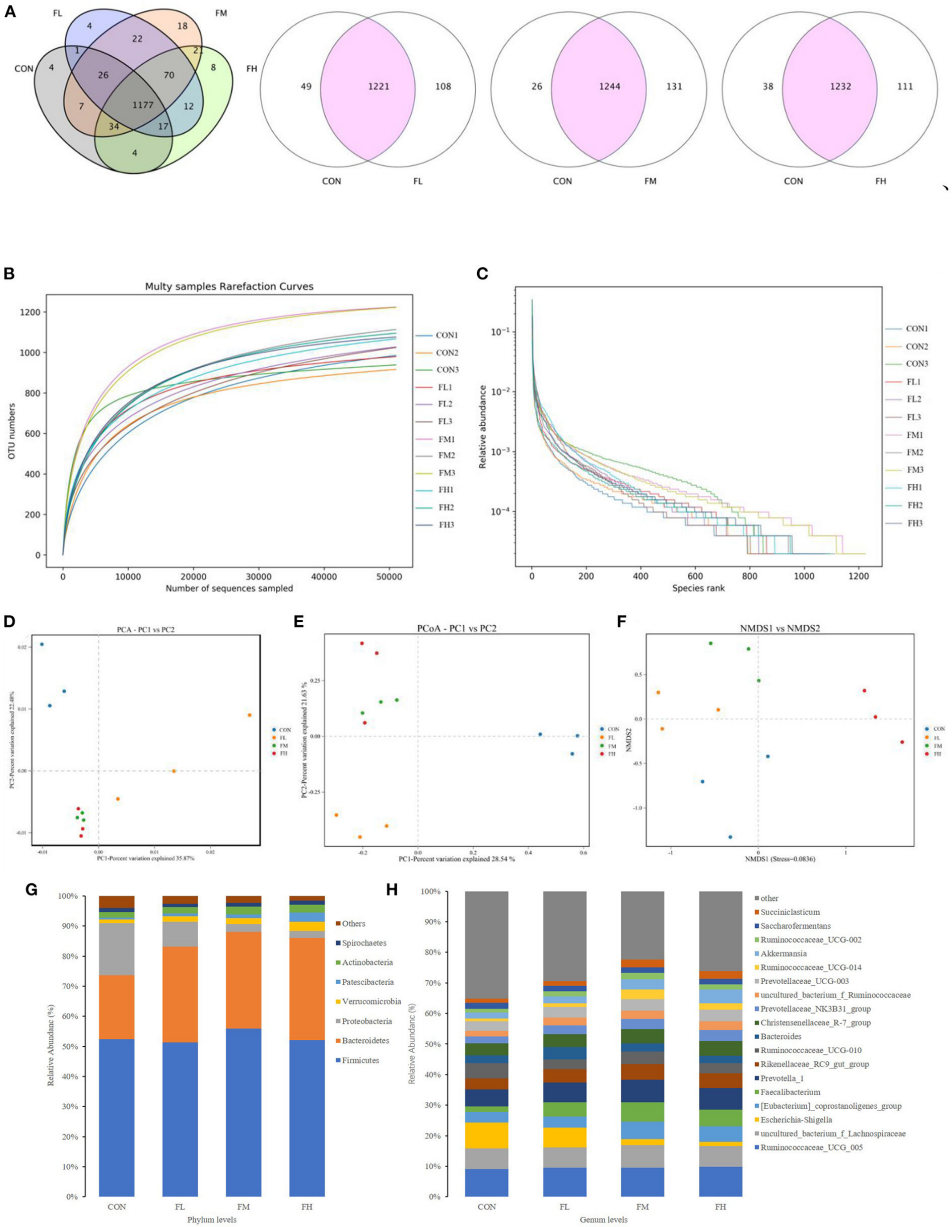
Effects of fucoidan on the cecal microbiota of weaned lambs (*n* = 3). **(A)** Venn diagram. **(B,C)** Correlation curves of species diversity. Multi-sample rarefaction curves **(B)** and the Rank-Abundance curves **(C)**. **(D–F)** Bate diversity analysis. **(D)** MetaStats analysis-based PCA analysis; **(E)** PCoA analysis using weighted UniFrac distances; **(F)** NMDS analysis. **(G,H)** Histograms showing the abundance of microbiota in the cecum at the phylum **(left)** and genus **(right)** levels. CON, control; FL, 0.1% fucoidan (DMI); FM, 0.3% fucoidan (DMI); FH, 0.6% fucoidan (DMI).

The quantity of common and unique OTUs amongst the four groups is summarized in the Venn diagram of the OTUs (Figure 7A). In the four treatment groups, given fucoidan at 0–0.6%, the number of unique OTUs was 4, 4, 18, and 6, respectively, showing a trend of first increasing and then decreasing. This is consistent with trends in the total OTUs.

As shown in Figure 7B, the curve began to plateau when the number of effective sequences reached 10,000. This implies that the quantity of sequencing did not result in an increase in the cecum microbiota and that there was sufficient coverage of almost all microbiota species in all samples, which indicates robust microbiological data. Moreover, the rank abundance curves displayed a smooth trend. A flatter slope indicates a more homogeneous distribution of species (Figure 7C). The abscissa span increased first and then decreased as the concentration of fucoidan was increased, and this trend showed that the species abundances increased first and then decreased as the fucoidan dose increased.

The Ace, Chao, Simpson, and Shannon indexes were used to determine the diversity and richness of the microbial communities in cecal samples (Table 2). Chao1 increased significantly with increasing fucoidan levels (*P* = 0.072). Fucoidan-fed groups also considerably outperformed the CON group in terms of Shannon and Simpson diversity indices (*P* < 0.05), but no other discrepancies were observed (fucoidan groups not shown). The gut microbial alpha-diversity of the weaned lambs was improved by supplementation with fucoidan, especially in the FM group.

**Table 2:** The effects of fucoidan on the microbial alpha diversity index in the ceca of weaned lambs.

**Items**	**Fucoidan levels**				**SEM**	***P*-value**
	**CON**	**FL**	**FM**	**FH**		
ACE	1082.24	1140.47	1099.44	1239.05	25.746	0.113
Chao1	1096.44	1144.36	1184.35	1244.07	21.763	0.072
Simpson	0.84^b^	0.95^a^	0.97^a^	0.95^a^	0.018	0.027
Shannon	4.95^b^	6.82^a^	7.15^a^	7.21^a^	0.339	0.023
Coverage/%	99.70	99.68	99.73	99.77	0.023	0.555

a,b,c Demonstrate a statistically significant distinction, *P* < 0.05.

To pinpoint the main microorganisms that significantly influenced the PCA findings, we computed the PCA values. The first three (PC1, PC2, and PC3) components accounted for 35.87, 22.48, and 7.95%, respectively, and the cumulative contribution rate reached 66.30% (*P* < 0.03) (Figure 7D). Compared with the control group, fucoidan-fed groups were clustered more closely together and had greater similarities. However, the dissimilarity in cecal microbial communities of the FL group was greater compared with the other groups. This suggests that fucoidan may introduce dramatic changes to the overall structure of the gut microbial community, but no dose effects were found. The PCoA was very consistent with the results of the PCA (Figure 7E). Non-metric multidimensional scaling (NMDS) analysis also demonstrated that the gut flora of the samples differed. NMDS analysis indicated that the microbiome composition among the four groups was considerably different (Stress = 0.0836) (Figure 7F). Collectively, these results showed that the fucoidan treatments affected the diversity and composition of the cecal microbiota of the weaned lambs.

Fucoidan added to milk replacers enhanced the relative abundance of Bacteroidetes and Actinobacteria in the cecum (*P* = 0.011 and 0.025, respectively) while decreasing the relative richness of Proteobacteria (*P* = 0.009) (Table 3 and Figure 7G). Fucoidan also raised the relative abundance of Rikenellaceae_RC9_gut_group, Prevotella_1, and Faecalibacterium in the cecum (*P* < 0.05), while decreasing the relative abundance of Escherichia-Shigella (*P* < 0.001). In this investigation, adding milk replacer supplemented with 0.3–0.6% fucoidan led to an increase in the relative abundance of Ruminococcaceae_UCG-014, Succiniclasticum, and Akkermansia in the cecum (*P* < 0.05) (Table 4 and Figure 7H).

**Table 3:** The effects of fucoidan on the major bacterial phyla in the compositions of the ceca (average relative abundance ≥1% in at least one group) (%).

**Items**	**Fucoidan levels**				**SEM**	***P*-value**
	**CON**	**FL**	**FM**	**FH**		
Firmicutes	52.35	51.25	55.86	52.05	1.131	0.546
Bacteroidetes	21.24^b^	31.91^a^	32.17^a^	33.96^a^	1.762	0.011
Proteobacteria	17.39^a^	8.29^b^	2.62^c^	2.31^c^	2.509	0.009
Actinobacteria	1.88^b^	2.01^b^	2.59^a^	2.69^a^	1.300	0.025
Verrucomicrobia	1.12	1.81	1.90	3.07	0.457	0.567
Patescibacteria	0.69	0.95	1.30	3.01	0.374	0.090
Spirochaetes	1.35	1.10	1.24	1.35	0.101	0.844

a,b,c Demonstrate a statistically significant distinction, *P* < 0.05.

**Table 4:** The effects of fucoidan on the major bacterial genera in the compositions of the ceca (average relative abundance ≥1% in at least one group) (%).

**Items**	**Fucoidan levels**				**SEM**	***P*-value**
	**CON**	**FL**	**FM**	**FH**		
Ruminococcaceae_UCG-005	8.59	9.17	9.22	9.46	0.290	0.807
Uncultured_bacterium_f_Lachnospiraceae	6.33	6.46	7.17	6.62	0.365	0.893
Escherichia-Shigella	7.87^a^	6.14^b^	1.87^c^	1.30^c^	0.859	< 0.001
(Eubacterium)_coprostanoligenes_group	3.32	3.50	5.62	4.91	0.382	0.063
Faecalibacterium	1.71^b^	4.51^a^	6.16^a^	5.28^a^	0.552	0.002
Prevotella_1	5.26^c^	6.26^b^	7.06^a^	6.91^a^	0.231	0.001
Rikenellaceae_RC9_gut_group	3.42^b^	4.25^a^	4.96^a^	4.69^a^	0.198	0.004
Ruminococcaceae_UCG-010	4.59	3.02	3.96	3.18	0.291	0.196
Bacteroides	2.46	3.85	2.72	2.38	0.253	0.130
Christensenellaceae_R-7_group	3.64	4.09	4.44	4.56	0.145	0.077
Prevotellaceae_NK3B31_group	2.09	2.80	3.29	3.51	0.213	0.057
Uncultured_bacterium_f_Ruminococcaceae	1.78	2.48	2.57	2.79	0.152	0.073
Prevotellaceae_UCG-003	2.98	3.32	3.64	3.65	0.112	0.081
Ruminococcaceae_UCG-014	0.87^c^	1.22^c^	3.06^a^	2.05^b^	0.273	0.001
Akkermansia	1.81^c^	2.09^c^	3.23^b^	4.40^a^	0.341	0.002
Ruminococcaceae_UCG-002	1.12	1.65	2.06	1.61	0.152	0.177
Saccharofermentans	1.86	1.77	1.74	1.71	0.024	0.116
Succiniclasticum	1.22^b^	1.33^b^	2.45^a^	2.52^a^	0.191	< 0.001

a,b,c Demonstrate a statistically significant distinction, *P* < 0.05.

## Discussion

Previous studies on lambs with non-specific pathogenic diarrhea found that body weight and respiratory frequency of diarrhea were significantly reduced after weaning compared to before weaning, and the serum cytokines IL-4, IL-6, and IL-8 were significantly higher than in healthy lambs (26). Hence, diarrhea after weaning can easily induce an inflammatory response in lambs. This is in line with the findings of other investigations (20). Our findings demonstrated that fucoidan improved the PWD of weaned lambs. Specifically, 0.3~0.6% fucoidan has an obvious inhibitory effect on diarrhea in early-weaned lambs, with the diarrhea rate reduced by more than 50%. Fucoidan is composed of fucose and sulfate groups, which are responsible for a variety of biological effects (11–18). Hence, we speculate that there are three possible reasons why fucoidan reduces the rate of diarrhea in lambs. First, due to the action of the esophageal groove, plant polysaccharide that has not been degraded by the rumen microorganisms flows through the hindgut, which stimulates the secretion of mucous substances by intestinal cells. For example, the secretion of digestive enzymes in the small intestine helps lambs digest (27) and absorb nutrients and the goblet cells in the colon secrete mucin (28, 29). This can prevent bacteria from directly contacting the intestinal epithelial cells and reduce the possibility of non-infectious diarrhea (30). Second, fucoidan increases the activity and secretion of intestinal antioxidant enzymes, which scavenge excessive intestinal free radicals and prevent free radical-related disorders of metabolism (31, 32). Studies have shown that fucoidan's antioxidant properties depend on the composition of its monosaccharides, the location and quantity of its sulfate groups, as well as its molecular weight (33–35). Additionally, there are three main mechanisms explaining its mode of action. ① Reactive oxygen species (ROS) are captured by lipid peroxidation, combining with hydroxyl ions on the hydrocarbon chain of the polysaccharide to create water molecules. Peroxy radicals are further oxidized to form compounds that the body can tolerate by the reaction of single-electron carbon atoms. In addition, single-electron carbon atoms are further oxidized into peroxy radicals, which are then transformed into biocompatible compounds (36). ② the -OH from polysaccharide rings complexes with metal ions to generate free radicals and indirectly scavenges ROS (37). ③ Conventional antioxidant functions involve increasing the activities of antioxidant enzymes, such as SOD and GSH-Px, and terminating free radical chain reactions (38). Meanwhile, fucoidan promotes the maturation of intestinal mucosal immunity and reduces chronic intestinal inflammation and repeated immune responses, thus reducing intestinal tissue damage (14). Finally, fucoidan promotes the proliferation and attachment of beneficial bacteria and forms a microorganism barrier, thereby inhibiting the colonization and reproduction of some pathogenic bacteria (39, 40). Therefore, this study further elaborates on the relieving effect of fucoidan on diarrhea in early-weaned lambs from the aspects of colon tissue morphology, antioxidant enzymes, cytokines, and cecal microbes.

The colon consists of the mucosa, submucosa, muscularis, and serosa. Important indexes for assessing the colon's absorption capacity are the villus height, crypt depth, and villus height/crypt depth ratio. Goblet cells are one of the intestinal mucosal epithelial cells. The number of goblet cells gradually increases from the small intestine to the large intestine. The mucus secreted by these goblet cells has lubricating and protective effects on the intestine, participates in intestinal mucosal immunity and intestinal injury surface reconstruction, and maintains the integrity of the mucosal barrier (41). According to our findings, the colonic mucosas in the fucoidan groups were thicker and the mucosal epithelium was more intact compared to the group that did not get fucoidan (Figure 3A). In the lamb colon, it was discovered that 0.3–0.6% fucoidan increased the number of goblet cells, villus height, and mucosal thickness. Additionally, the ratio of villus height to crypt depth and muscle layer thickness both significantly increased (Figures 3B,C), suggesting that fucoidan can treat intestinal health issues brought on by weaning stress. This is another plausible explanation for the decreased rate of diarrhea.

Both non-infectious and infectious diarrhea are sources of harmful stimuli, prompting intestinal tissue to produce a large amount of ROS and their metabolites, leading to a redox imbalance in the body. This will exacerbate potential hazards such as lipid peroxidation, DNA damage, and inflammatory cytokine overexpression, inducing the apoptosis of mucosal cells. Chronic inflammation can also lead to metabolic disorders of the body and cause diseases (42–44). Therefore, the increase in lipid oxidation products and immune activity may explain the increased diarrhea rate and colonic mucosal damage in early-weaned lambs. Our results showed that feeding fucoidan increased T-AOC and the activities of SOD and GSH-Px in the colon, and decreased MDA content. Fucoidan may also stimulate the Nrf2/ARE signaling pathway in the small intestine to improve its cytoprotective effects (16, 45). Hence, fucoidan promotes the synthesis and secretion of antioxidant enzymes, effectively removes excessive ROS, and relieves the damage to tissues caused by oxidative stress (46, 47). In contrast, feeding 0.3–0.6% fucoidan lowered colonic IgM content in comparison to the control group, which was compatible with serum immunity results (20). Meanwhile, fucoidan treatment decreased the content of colonic pro-inflammatory factors (IL-1β, TNF-α, and INF-γ) and increased the content of the anti-inflammatory factor IL-10, compared with the control group without fucoidan. In particular, feeding 0.3% fucoidan increased colonic IgA, IgG, and SIgA contents. This is in line with findings by Walsh et al. (48) and Aikahtane et al. (49). The changes in these immunoglobulin contents indicated that the weaned lambs had been recently infected by pathogenic bacteria, resulting in the increased immune response. Many studies have shown that fucoidan maintains the intestinal immune balance by regulating the ratio of Th1/Th2 and the expression of immunoglobulins in intestinal helper T-cells (14, 16, 18). By activating the NF-κB, MAPK, and AP-1 signaling pathways, fucoidan induces the production of NO, TNF-α, IL-1β, and other cytokines to regulate intestinal immunity (50, 51). Similarly, Liying et al. (52) showed that the lipopolysaccharide-induced production of TNF-α and IL-1β in RAW 264.7 macrophages was significantly inhibited by fucoidan extracted from *Laminaria japonica* (without sulfate groups at the C-4 and C-2 sites), resulting in less cell death. In short, fucoidan increased the levels of intestinal antibodies in early-weaned lambs, improved the integrity of the intestinal barrier, and alleviated weaning stress and intestinal inflammation. However, most studies that have hinted at the mechanisms by which fucoidan affects the immune system have been unable to explain how its unusual structure and chemical make-up serve a specific functional purpose (53, 54). In the future, our research team plans to investigate the role of fucoidan in the immune system and antioxidant defenses of goat intestinal epithelial cells.

Prior to weaning, the gastrointestinal tract (GIT) of young ruminants plays a vital role in nutrient absorption, similar to piglets. However, symptoms of weaning stress are often accompanied by disorders of the gut microbiota (55, 56). The GIT microbiota is an important “invisible organ” in the body (57). Antagonism is a crucial mechanism for maintaining the microecological balance in the gut, including attachment space and/or nutrients, producing antibacterial substances, and induced immunity (56). Our 16S rDNA gene sequencing results found that the Simpson and ACE indexes were significantly increased in the fucoidan-fed groups compared with the control group. Therefore, the addition of fucoidan in milk substitute increased the bacterial richness and diversity of the colon in weaning lambs. The diversity of microorganisms helps to maintain the stability and resistance of the intestinal ecosystem, which in turn assists in the maintenance of intestinal health and reduces the risk of disease (58). Firmicutes, Proteobacteria, Bacteroidetes, and Actinobacteria were the dominant bacterial phyla. Fucoidan (0.3~0.6%) supplementation significantly increased the relative abundance of Bacteroidetes and Actinobacteria, but decreased that of Proteobacteria. Furthermore, there was a significant increase in the relative abundances of 9 genera (Firmicutes: Romboutsia, Ruminococcus_1, Ruminococcaceae_NK4A214_group, Ruminocaceae_UCG-014, Faecalibacterium, and Succiniclasticum; Bacteroidetes: Prevotella_1, Rikenellaceae_RC9_gut_group, and Prevotellaceae). These genera selectively promote the degradation of saccharides (59, 60) and crude fiber (61). By reducing intestinal pH and thus limiting the colonization of harmful bacteria that are sensitive to low pH, organic acids created by the fermentation of feed can prevent pathogenic infection (62). Meanwhile, in the gut, SCFA acts as a signal molecule that regulates a versatile class of intestinal immune cells (63, 64). Crucially, SCFA can maintain the metabolic equilibrium of colon-forming cells and protect them from outside harm (65). Our results demonstrated that cecal propionic acid, butyric acid, and total volatile fatty acid content were enhanced by fucoidan feeding. Fucoidan increased the abundance of SCFA-producing bacteria in the gut, thereby promoting the production of SCFA in the cecum, in line with the findings of Xue et al. (39). In addition, fucoidan reduced the abundance of Proteobacteria phyla, especially Escherichia-Shigella abundance. According to Liu Mengjian et al. (21) and Liu et al. (66), low molecular-weight fucoidan improves the composition and diversity of the gut microbiota in weaned lambs (39). In particular, fucoidan efficiently inhibits Escherichia-Shigella growth (66). Fucoidan also ameliorates intestinal damage in weaned lambs by boosting neutrophil and macrophage numbers (67) and lowering inflammatory cytokine expression (68). This could also partly explain the above-mentioned results of the decreased fecal index, diarrhea rate, and cytokine content in the colons of lambs fed fucoidan in the current study. The relative abundance of Succiniclasticum and Akkermansia in the cecum was also raised by adding milk replacer supplemented with 0.3–0.6% fucoidan. The relative abundance of Succiniclasticum and Akkermansia in the cecum is much lower than that of other bacteria, but the steady-state effect on the gastrointestinal environment is far greater than the numerical difference (69–72).

## Conclusion

Collectively, the results presented herein suggest that milk replacement supplementation with 0.3–0.6% (DMI) fucoidan could decrease diarrhea rates. Fucoidan may be closely linked with improvements in the colon antioxidant capacity and immunity, as well as the gut microbiota community composition in weaned lambs. The results of this study provide a scientific basis for the application of fucoidan as a feed additive for protecting intestinal health and performance in weaned lambs.

## Data availability statement

The original contributions presented in the study are included in the article/supplementary material, further inquiries can be directed to the corresponding authors.

## Ethics statement

The animal study was reviewed and approved by Guangdong Ocean University's Animal Care and Use Committee. Written informed consent was obtained from the owners for the participation of their animals in this study.

## Author contributions

FY and GG: conceptualization. GG: data curation and editing the manuscript. FY, RL, and HY: methodology and writing—review and editing. ZW and CF: software. WY: investigation. FY, SG, and ZG: resources. FY: project administration and funding acquisition. All authors have read and agreed to the published version of the manuscript.

## Funding

This research was funded by the Fundamental Research Funds for the Guangdong Province (2020A03025), the Fundamental Research Funds for the Agriculture and Rural Affairs of Guangdong Department (2019KJ127), the Fundamental Research Funds for Education Department of Guangdong (2021003), and the Fundamental Research Funds for Exploration and promotion of goat standardized breeding mode of Qingyuan (200805164563031).

## Conflict of interest

The authors declare that the research was conducted in the absence of any commercial or financial relationships that could be construed as a potential conflict of interest.

## Publisher's note

All claims expressed in this article are solely those of the authors and do not necessarily represent those of their affiliated organizations, or those of the publisher, the editors and the reviewers. Any product that may be evaluated in this article, or claim that may be made by its manufacturer, is not guaranteed or endorsed by the publisher.
